# Ultra-sustainable Fe_78_Si_9_B_13_ metallic glass as a catalyst for activation of persulfate on methylene blue degradation under UV-Vis light

**DOI:** 10.1038/srep38520

**Published:** 2016-12-06

**Authors:** Zhe Jia, Xiaoguang Duan, Wenchang Zhang, Weimin Wang, Hongqi Sun, Shaobin Wang, Lai-Chang Zhang

**Affiliations:** 1 School of Engineering, Edith Cowan University, 270 Joondalup Drive, Joondalup, Perth, WA 6027, Australia; 2 Department of Chemical Engineering, Curtin University, GPO Box U1987, Perth, WA 6845, Australia; 3 Environmental Protection Administration of Ji’an City, Ji’an, Jiangxi Province, 343000, China; 4 School of Materials Science and Engineering, Shandong University, Jinan, Shandong 250061, China

## Abstract

Stability and reusability are important characteristics of advanced catalysts for wastewater treatment. In this work, for the first time, sulfate radicals (SO_4_∙^−^) with a high oxidative potential (E^o^ = 2.5–3.1 V) were successfully activated from persulfate by a Fe_78_Si_9_B_13_ metallic glass. This alloy exhibited a superior surface stability and reusability while activating persulfate as indicated by it being used for 30 times while maintaining an acceptable methylene blue (MB) degradation rate. The produced SiO_2_ layer on the ribbon surface expanded strongly from the fresh use to the 20^th^ use, providing stable protection of the buried Fe. MB degradation and kinetic study revealed 100% of the dye degradation with a kinetic rate *k* = 0.640 within 20 min under rational parameter control. The dominant reactive species for dye molecule decomposition in the first 10 min of the reaction was hydroxyl radicals (∙OH, E^o^ = 2.7 V) and in the last 10 min was sulfate radicals (SO_4_∙^−^), respectively. Empirical operating variables for dye degradation in this work were under catalyst dosage 0.5 g/L, light irradiation 7.7 μW/cm^2^, and persulfate concentration 1.0 mmol/L. The amorphous Fe_78_Si_9_B_13_ alloy in this work will open a new gate for wastewater remediation.

Catalysis has greatly enhanced removal of toxic pollutant combinations in effluents, such as dyes^1^, heavy metals^2^, phenols^3^ and nitrates^4^. Among current catalytic processes, researchers have strongly focused on the advanced oxidation processes (AOPs) due to their superior degradation and mineralization efficiency with hazardous compounds in wastewater^5^. Compared to alternatives, such as nano-filtration^6^, adsorption^7^, flocculation^8^, ion exchange^9^ and biological degradation^10^, AOPs do not require further treatment and can completely mineralize organic contaminants in wastewater. From economic and chemical perspectives, AOPs can efficiently degrade most of organic pollutants to non-toxic and ubiquitous substances, such as CO_2_, H_2_O and low molecular weight aliphatic acids^11^,^12^,^13^,^14^,^15^.

Emerging AOPs show promise for producing highly reactive transitory species such as sulfate radicals (SO_4_∙^−^, E^o^ = 2.5–3.1 V^16^) for remediating toxic components in the aqueous matrix. Compared to hydroxyl radicals (∙OH, E^o^ = 2.7–2.8 V^17^), SO_4_∙^−^ demonstrates a superiority on account of high redox potential, large pH range and free secondary pollutant^18^. Because of the strong redox potential and stability, persulfate (S_2_O_8_^2−^, E^o^ = 2.01 V^19^) is a promising source of SO_4_∙^−^. The established methods for easily activating S_2_O_8_^2−^ to SO_4_∙^−^ are sole activation by UV^20^ or heat^21^, and catalytic activation by metal-free catalyst^22^ or metal-based catalyst^23^,^24^,^25^. Among these methods, the production rate of homogeneous activation of SO_4_∙^−^ by heat (*k* = 1.0 × 10^−7^ M^−1^s^−1 ^19) is relatively lower compared to catalytic activation. Recent studies indicate that heterogeneous catalytic activation of SO_4_∙^−^ using iron can significantly enhance the production rate to *k* = 2.0 × 10^1^ M^−1^ s^−1 ^19. Iron-based catalyst covers a range from the homogeneous to the heterogeneous methods and has been extensively reported, such as ferric or ferrous sulphate for homogeneous reaction^26^ and goethite (α-FeOOH)^27^, magnetite (Fe_3_O_4_)^28^, hematite (α-Fe_2_O_3_)^29^, maghemite (g-Fe_2_O_3_)^30^ and zero-valent iron (ZVI)^31^,^32^,^33^ for heterogeneous reaction. Therefore, an appropriate Fe-based catalyst that features environmentally friendly and sustainable development is highly demanded in wastewater remediation.

The traditional homogeneous method focuses on ferrous or ferric to produce strong oxidative radicals^34^, but has some inherent restrictions, such as being less efficient and reusable, thus creating a secondary sludge disposal problem^35^,^36^. Heterogeneous Fenton method using zero-valent iron (ZVI) is an alternative technology for water purification^37^. Based on its magnetic property, ZVI is easily recycled and therefore the secondary sludge is effectively reduced. However, as dye wastewater is normally at a high temperature, the oxidization property of ZVI is very constrained^38^. Moreover, the ZVI would lose activity once active sites are occupied by the oxidants during water purification^38^. Fe-based amorphous alloys are a special type of ZVI materials^39^, and the ultrafast degradation and mineralization efficiency of hazardous contaminants in the wastewater matrix have been achieved by using Fe-B^40^, Fe-Si-B^13^, Fe-Mo-Si-B^41^, Fe-Si-B-Cu-Nb^42^ and Fe-Nb-Si-B^43^ alloys. Due to their superior soft magnetic property and chemical stability, Fe-based metallic glasses are readily recycled and have an acceptable level of surface decay, thus offering the potential for dramatic cost reductions when employed as a catalyst in industrial settings^13^,^40^,^41^,^42^,^43^.

This paper involves an original approach in which the potential of a Fe-based amorphous alloy with a nominal composition of Fe_78_Si_9_B_13_, is explored as a catalyst for activating persulfate to produce sulfate radicals (SO_4_∙^−^). Reusability issues, including conductivities and iron-leaching for amorphous Fe_78_Si_9_B_13_ ribbon under optimum experimental conditions, are studied in detail. The effect of various parameters, including persulfate concentration, Fe_78_Si_9_B_13_ ribbon dosage and irradiation intensity, on the dye degradation efficiency is also investigated. In addition, the mechanisms for long-life reusability and surface stability of the fresh and used Fe_78_Si_9_B_13_ catalysts are also characterized.

## Results

### Characterization of amorphous Fe_78_Si_9_B_13_ ribbons

**Figure 1:**
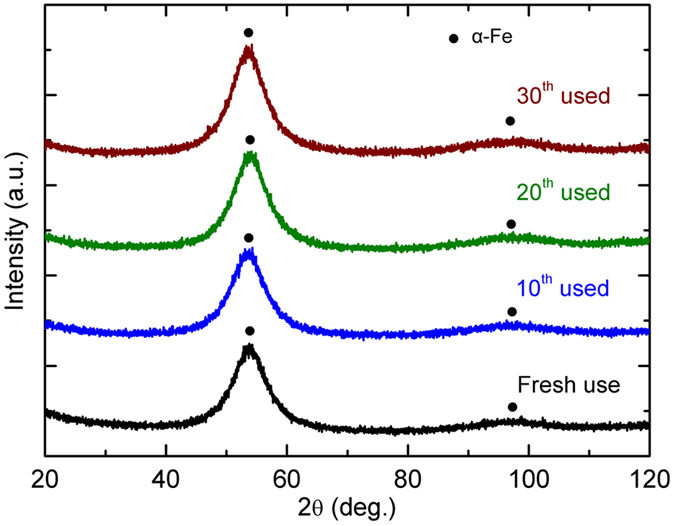
XRD patterns of fresh and used Fe_78_Si_9_B_13_ ribbons after multiple runs (Fe_78_Si_9_B_13_ dosage: 2.0 g/L, persulfate concentration: 1.0 mM, irradiation intensity: 7.7 μW/cm^2^, dye concentration: 20 ppm).

Figure 1 presents the XRD curve of fresh and used Fe_78_Si_9_B_13_ ribbons after 10, 20, and 30 runs. All ribbons exhibit a broad hump in the range of 2θ = 40–60°, indicating that the structures of the ribbons are predominantly in amorphous states^44^,^45^,^46^,^47^,^48^,^49^. Notably, the diffraction intensities at 2θ_*max*_ = 53.2° gradually increase with increasing the use times of ribbons, from 1172 counts for fresh use to 1546 counts for 30^th^ used ribbon, considering the same amount of ribbons were used for XRD characterization. This result indicates that α-Fe is gradually crystallized during the dye degradation under UV-Vis light, as observed in our previous work^13^.

**Figure 2:**
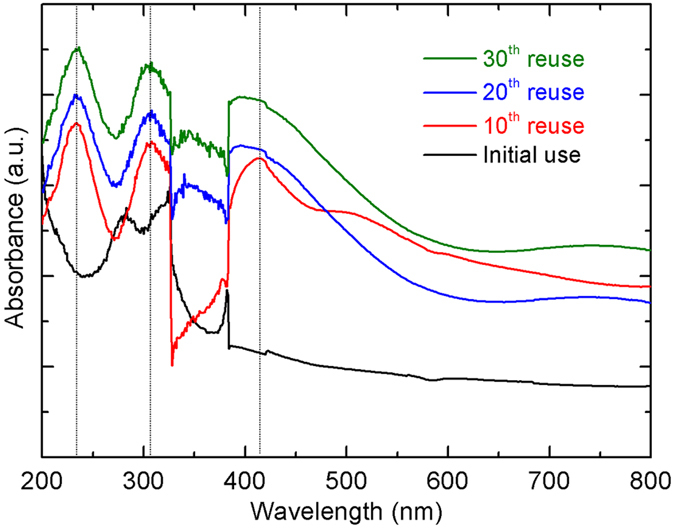
UV-DRS of the fresh and used amorphous Fe_78_Si_9_B_13_ ribbons.

**Figure 3:**
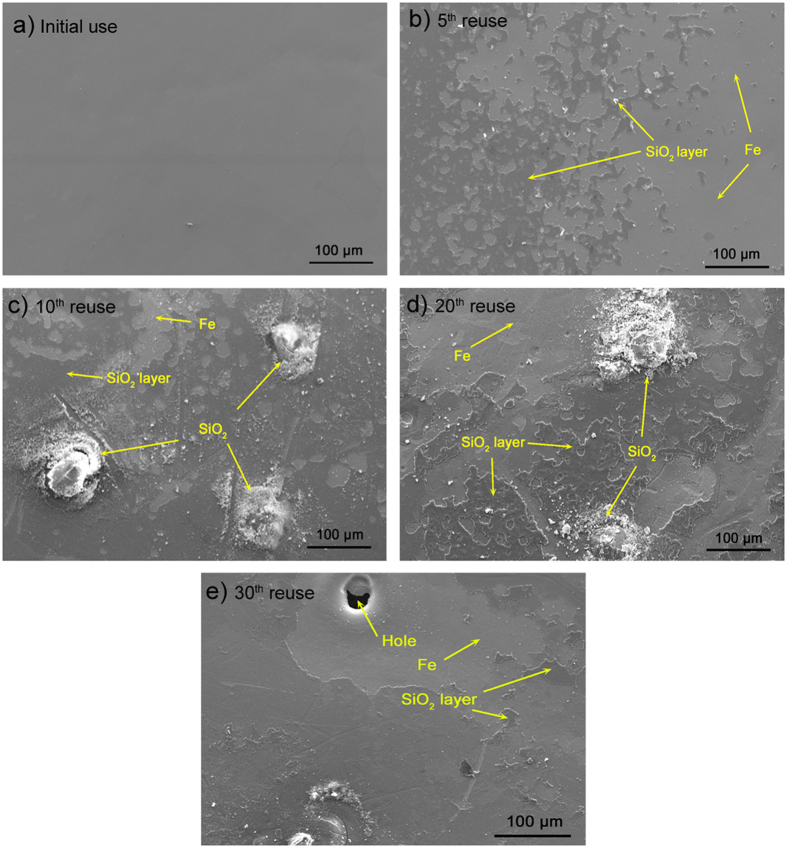
SEM micrographs of (**a**) fresh use, (**b**) 5^th^ use, (**c**) 10^th^ use, (**d**) 20^th^ use and (**e**) 30^th^ use of Fe_78_Si_9_B_13_ ribbons.

To further analyze the mechanism of reusability of Fe_78_Si_9_B_13_ ribbon during dye degradation, UV-DRS characterization is shown in Fig. 2. Compared to the fresh Fe_78_Si_9_B_13_ ribbon, all used ribbons present three obvious absorbance peaks at λ = 240 nm, 305 nm and 410 nm. This result indicates that the iron species (tetrahedrally or octahedrally coordinated) are still distributed homogeneously and new iron oxides are formed on the used Fe_78_Si_9_B_13_ ribbons during dye degradation^50^,^51^. The changes of surface morphology for the fresh and used Fe_78_Si_9_B_13_ ribbons are shown in Fig. 3. The free surface of the fresh ribbon is smooth (Fig. 3a), indicating no porosity or defects on that state. After the 5^th^ use (Fig. 3b), parts of the ribbon’s surface precipitate a SiO_2_ layer covering the buried Fe. In Fig. 3c, the SiO_2_ particles with an increased covering area of the SiO_2_ layer are first observed on the surface of the 10^th^ use ribbons. Increasing the use times from 10 to 20, enhances both the size of the SiO_2_ particles and the coverage area of SiO_2_ layer (Fig. 3d). However, the percentage of the SiO_2_ layer on the 30^th^ use ribbon is remarkably reduced, leading to the formation of holes (Fig. 3e).

### Surface stability and reusability of amorphous Fe_78_Si_9_B_13_ ribbon

To further analyze the significant effect on the surface stability of Fe_78_Si_9_B_13_ alloy, the results of reusability (Figure S1 in Supplementary) using the same ribbons were carried out under same parameters as follows: dye concentration of 20 ppm, irradiation intensity of 7.7 μW/cm^2^, Fe_78_Si_9_B_13_ dosage of 2.0 g/L, and persulfate concentration of 1.0 mM. It is confirmed that the persulfate can be activated to SO_4_•^−^ by UV-Vis light or heat^20^,^21^. As seen in Figure S1a and b, the MB decolor rate and reaction rate (*k*) by using the only persulfate are much lower compared to the addition of Fe_78_Si_9_B_13_ ribbons, as only 70.2% color removal and *k* = 0.092 were achieved after 20 min light irradiation. Increasing multiple cycles of used Fe_78_Si_9_B_13_ ribbons from fresh use to 30^th^ use, the kinetic rates (*k*) led to an acceptable decrease from k = 0.718 to k = 0.217 (*k* is still much higher than only using persulfate). Compared with our previous report^13^ of Fe_78_Si_9_B_13_ activation for H_2_O_2_, the reusability of Fe_78_Si_9_B_13_ ribbons is much enhanced by the activation for persulfate in this work.

**Figure 4:**
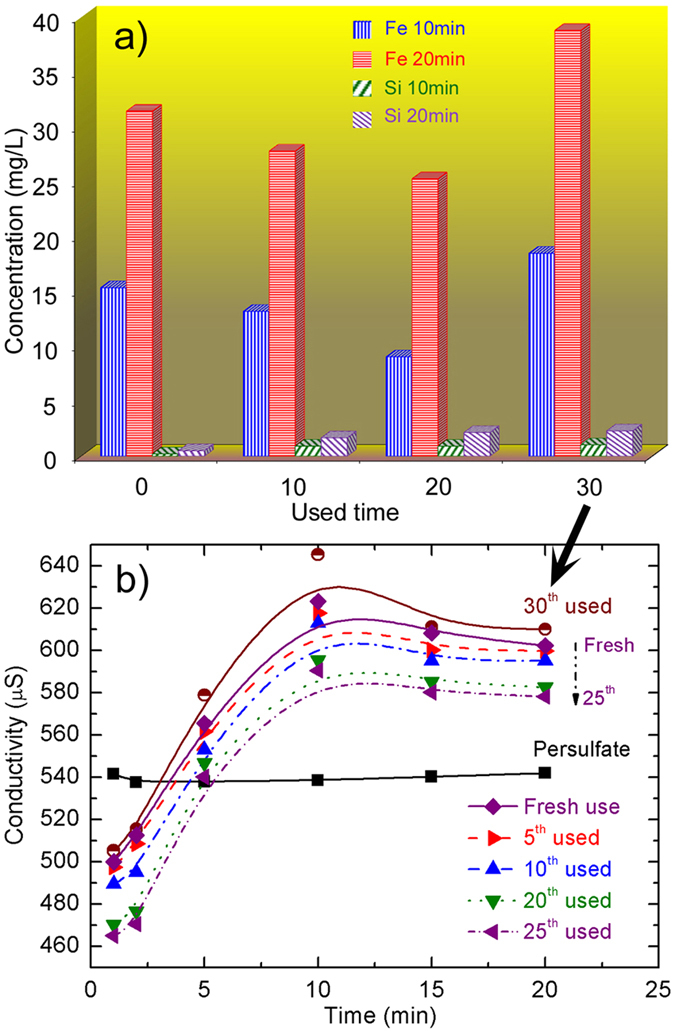
Changes in (**a**) Fe, Si concentrations and (**b**) conductivities in the sample solution with used Fe_78_Si_9_B_13_ ribbons under UV-Vis light (dye concentration: 20 ppm, irradiation intensity: 7.7 μW/cm^2^, Fe_78_Si_9_B_13_ dosage: 2.0 g/L, persulfate concentration: 1.0 mM).

Reusability is a particularly valuable property for amorphous Fe_78_Si_9_B_13_ alloy^52^,^53^. To study the mechanism for such high reusability, concentrations of leached Fe and Si as well as conductivity changes at various use times in the sample solution are shown in Fig. 4a and b. As seen in Fig. 4a, the concentration of leached Fe decreases from 31.5 mg/L for the fresh use of a ribbon to 25.3 mg/L for 20^th^ use of the ribbon and then to 38.8 mg/L for the 30^th^ use ribbon at 20 min. Moreover, the conductivity decreases from fresh use to the 25^th^ use and then increases again at the 30^th^ use at 20 min (Fig. 4b). These results indicate that the iron on the Fe_78_Si_9_B_13_ surface is gradually overlapped by the precipitated SiO_2_ layer (Fig. 3) during the dye degradation under UV-Vis light in the 25 use times. The lower rate of Fe leaching reduces the conductivity of the sample solution. Accordingly, the dye degradation rate reduces (Figure S1) because the persulfate is activated by the ferrous in the solution rather than the solid zero-valent iron in the ribbon^19^,^54^.

**Figure 5:**
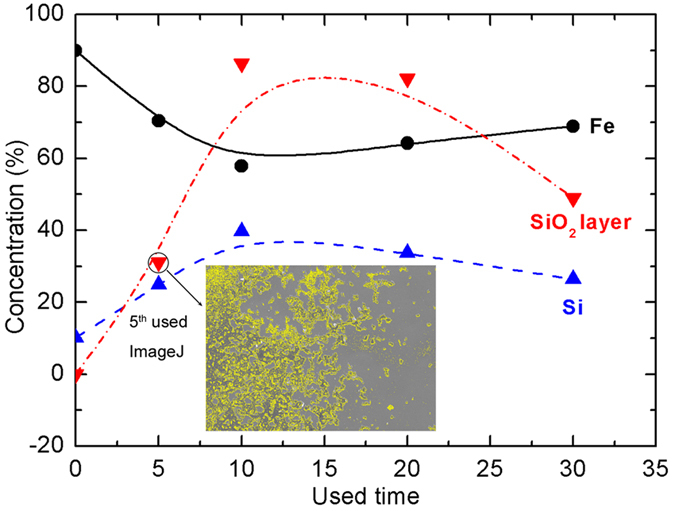
Changes of Fe, Si and SiO_2_ layer percentages for various used Fe_78_Si_9_B_13_ ribbons (inset, SiO_2_ layer percentage for 5th use ribbons, surface fitted by imageJ).

Elemental distribution on the Fe_78_Si_9_B_13_ ribbon surface is another significant indicator of material stability and reusability. Figure 5 shows the comparative results of the changes of Fe, Si and SiO_2_ layer (inset, fitted by imageJ for the 5^th^ use) for fresh and various use times for Fe_78_Si_9_B_13_ ribbons. The initial atomic ratio of Fe to Si before the dye degradation is 89.9%:10.1% (9:1), which agrees well with the nominal atomic proportion of 78:9 in the Fe_78_Si_9_B_13_ alloy. Increasing the use times of Fe_78_Si_9_B_13_ ribbons from fresh use to 20^th^ use sharply reduces the Fe atomic percentage from 89.9% to 64.2% on the surface, whereas the Si atom and SiO_2_ layer percentages increase from 10.1% to 33.7% and 0% to 82.1%, respectively. These results confirm that the SiO_2_ layer gradually formed on the ribbon surface, effectively protects against Fe leaching and maintains acceptable dye degradation efficiency (Figure S1).

### Methylene blue degradation

**Figure 6:**
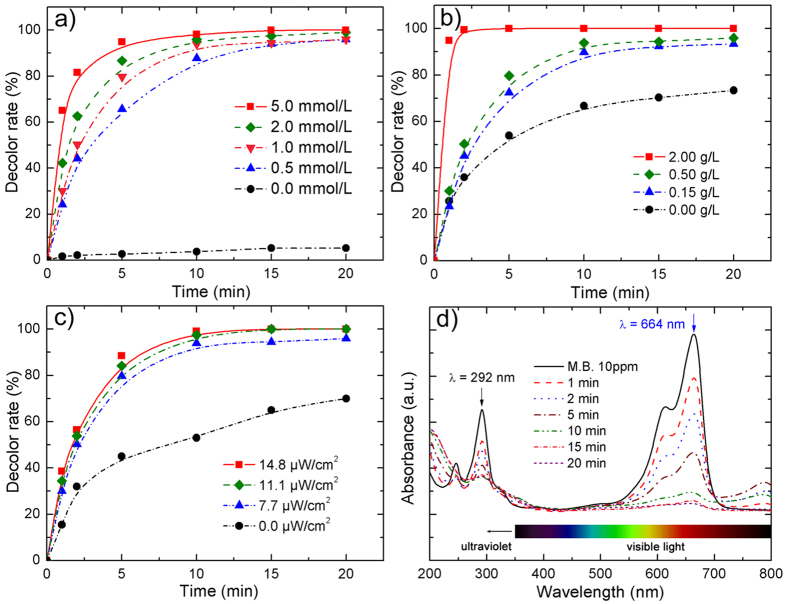
Color removal as a function of (**a**) persulfate concentration, (**b**) Fe_78_Si_9_B_13_ dosage, (**c**) light intensity and (**d**) UV-Vis spectrum at different time intervals (if not mentioned, the reaction conditions are irradiation intensity: 7.7 μW/cm^2^, dye concentration: 20 ppm, Fe_78_Si_9_B_13_ dosage: 0.5 g/L, persulfate concentration: 1.0 mM).

Amorphous Fe_78_Si_9_B_13_ alloy has a superior efficiency for activation of persulfate in MB dye degradation. Compared to our previous work with H_2_O_2_^13^, there is no further consideration of pH with the addition of persulfate. Figure 6 shows MB dye degradation under various parameters and the corresponding reaction rates (*k*) are summarized in Table 1. Clearly, the MB dye degradation rate is significantly impacted by persulfate concentration (Fig. 6a), Fe_78_Si_9_B_13_ dosage (Fig. 6b) and irradiation intensity (Fig. 6c).

As seen in Fig. 6a, solely using Fe_78_Si_9_B_13_ cannot effectively degrade MB dye as the oxidative radicals cannot be produced without adding persulfate. Increasing the persulfate concentration from 0.0 mM to 5.0 mM dramatically enhances the reaction rate from *k* = 0.002 to *k* = 0.658 (Table 1) as it ensures that sufficient SO_4_•^−^ and ∙OH can be activated, corresponding to the following Equation (1) – (3):


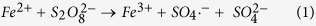



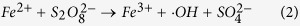



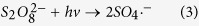


The production of SO_4_∙^−^ (E^o^ = 2.5–3.1 V) and ∙OH (E^o^ = 2.70–2.80 V) can be used for rapid MB dye degradation^55^,^56^. The reaction rates (*k*) from 0 mM to 1.0 mM are also markedly enhanced from *k* = 0.002 to *k* = 0.626 compared to the slight improvement from 1.0 mM (*k* = 0.626) to 5.0 mM (*k* = 0.658). For an economic perspective, 1.0 mM persulfate will be used in future work. The effect of Fe_78_Si_9_B_13_ dosage is also important (Fig. 6b). The SO_4_∙^−^ radicals are able to be produced by using persulfate only under UV-Vis light albeit at a relatively low dye degradation reaction rate (*k* = 0.092). Increasing the Fe_78_Si_9_B_13_ dosage from 0.0 g/L to 0.5 g/L can effectively enhance the reaction rates from *k* = 0.092 to *k* = 0.640. However, only slight improvement of the reaction rate is observed with a further increase in catalyst dosage from 0.5 g/L to 2.0 g/L. Clearly, the production rate of SO_4_∙^−^ radicals can be strongly enhanced by adding a moderate amount of Fe-based catalyst^19^. Furthermore, the dye degradation efficiency is also significantly impacted by light irradiation intensity (Fig. 6c). The reaction rate without light irradiation is only *k* = 0.228, whereas increasing the light intensity to 7.7 μW/cm^2^ provides a dramatical improvement to *k* = 0.640. Increasing the light intensity obviously can enhance the production of SO_4_∙^−^ radicals. Moreover, the effect on ferrous production can be significantly enhanced according to the Equation 4:


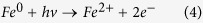


Figure 6d shows the UV-Vis spectra of MB dye degradation for different periods of UV-Vis light irradiation. The absorbance peaks of MB dye are characterized at λ = 292 nm and λ = 664 nm, indicating the triazine group (π–π* transition) and heteropoly aromatic linkage (including chromophore and auxochrome (-CH_3_)), respectively. The peak at λ = 664 nm is invisible after 20 min light irradiation, suggesting that the presence of color has been totally removed. The peak located at λ = 292 nm also decreases gradually along the irradiation time, which indicates that the final products are H_2_O, CO_2_, NO_3_^−^, and SO_4_^2− ^14. Figure S2 shows the visible color changes of MB dye in the presence of only Fe_78_Si_9_B_13_ ribbon, only persulfate, and persulfate coupled with Fe_78_Si_9_B_13_ ribbon. Clearly, the combined utilization of Fe_78_Si_9_B_13_ ribbon and persulfate has significantly enhanced the dye decolor rate under UV-Vis light, which is in good agreement with the aforementioned results.

**Figure 7:**
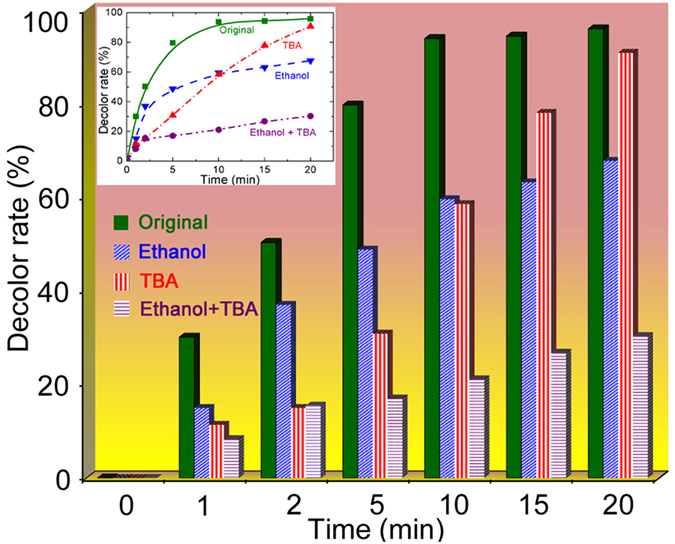
Comparable results of decolor rates (%) of catalysts with and without adding quenching agents of TBA (0.5 M) and Ethanol (0.5 M), with inset showing the line graph (irradiation intensity: 7.7 μW/cm^2^, dye concentration: 20 ppm, Fe_78_Si_9_B_13_ dosage: 0.5 g/L, persulfate concentration: 1.0 mM).

To study the dominant radicals for MB dye degradation, the changes in MB dye decolor rates were investigated after adding quenching agents of tertiary butanol (TBA 0.5 M) and ethanol (EtOH 0.5 M), which are normally used for quenching the produced ∙OH^57^,^58^ and SO_4_∙^− ^3, respectively. As shown in Fig. 7, the MB decolor rate decreases sharply after adding 0.5 M TBA or EtOH. The addition of TBA markedly reduces the dye decolor rate in first 10 min but the final decolor rate is approximately the same as the original result, whereas the sharp decrease in dye decolor rate after adding EtOH is concentrated in the last 10 min. Such a performance indicates that ∙OH is the dominant species for degradation of MB dye molecules during the first 10 min and SO_4_∙^−^ has more effect on the dye removal in the last 10 min. Moreover, the addition of both TBA and EtOH causes a significant reduction in the dye degradation efficiency from 100% to 30% which includes part of dye removal solely caused by the Fe_78_Si_9_B_13_ catalyst in Fig. 6a, demonstrating the significant effect of ∙OH and SO_4_∙^−^ on dye molecule decomposition.

## Discussion

The superior surface stability of amorphous Fe_78_Si_9_B_13_ alloy during MB degradation is attributed to the inclusion of Si atom (in the form of SiO_2_ layer), which could effectively reduce Fe leaching during dye degradation. Parts of the SiO_2_ layer would agglomerate to form SiO_2_ particles and finally fall from the surface as the dye solution is stirred. After the 30^th^ use, Si atoms in Fe_78_Si_9_B_13_ alloy are gradually consumed causing the SiO_2_ layer to decrease and the buried Fe to appear. These Fe would be continuously consumed by the activation of persulfate and further corroded as holes on the ribbon surface. The improved performance of the Fe_78_Si_9_B_13_ reusability is owing to the following reasons. (1) The pH value plays a significant role in the Fenton/Fenton-like reaction^59^,^60^, as hydroxyl radicals (∙OH) can be rapidly activated under pH 2 by using Fe_78_Si_9_B_13_ and H_2_O_2_^13^ whereas the pH of MB dye solution after adding Fe_78_Si_9_B_13_ and persulfate in this work is only 3.39. The acidic conditions can enhance the corrosive speed of the SiO_2_ layer on the Fe_78_Si_9_B_13_ surface leading to fast surface decay behaviour. (2) The SiO_2_ layer is progressively formed (Fig. 3) by using Fe_78_Si_9_B_13_ and persulfate, resulting in a stable protective environment for the active Fe atoms in the ribbon.

The results of Fe, Si leaching during the MB degradation further proves the surface aging behaviour on Fe_78_Si_9_B_13_ ribbons surface. The increase in the leached Fe and conductivity at 30^th^ use time is attributed to the formed SiO_2_ being gradually removed by the Vortex-stirrer, which results in more isolated iron on the ribbon surface exposure to the dye molecules thereby causing excessive iron leached. The concentration of leached Si in the solution is evidence of the removal of SiO_2_ (Fig. 4a). Notably, all the conductivity values for added Fe_78_Si_9_B_13_ ribbons are lower than for the only adding persulfate treatment in the first 5 min (Fig. 4b). This is owing to the dye degradation performance by using amorphous Fe_78_Si_9_B_13_ alloy in the initial 5 min is a pre-adsorption phase and further evidences that the dye molecule decomposition is based on a surface-reaction. At atomic level, increasing the use time from 20^th^ to 30^th^ results in an increase of Fe atoms and a decrease of Si atoms (in the form of SiO_2_) on the ribbon surface (Fig. 5), indicating that the 30^th^ use time is the threshold for “exhausting” the Fe_78_Si_9_B_13_ ribbon as the insufficient SiO_2_ layer cannot protect against further the Fe leaching, which may cause secondary pollution in the treated dye solution.

**Figure 8:**
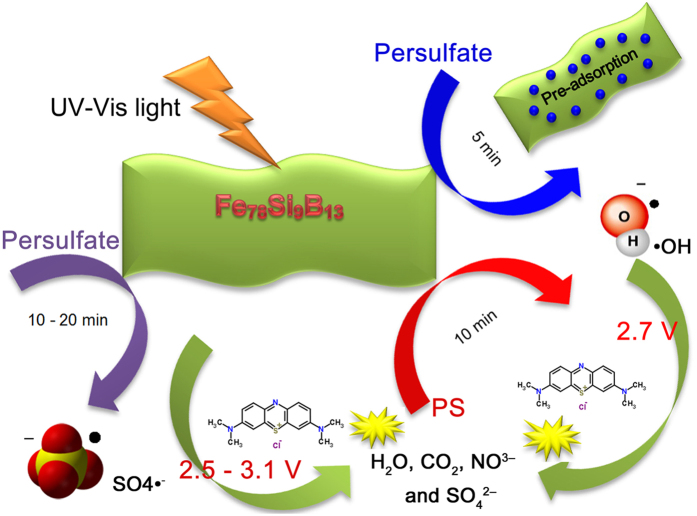
Amorphous Fe_78_Si_9_B_13_ alloy activation of persulfate and its dye degradation reaction.

To further highlight the mechanism of persulfate activation by Fe_78_Si_9_B_13_ under UV-Vis light, the result of generation processes of ∙OH and SO_4_∙^−^ is summarized in Fig. 8. According to Fig. 4b, dye molecules are pre-adsorbed on the ribbon surface in the first 5 min as indicated by the lower conductivity compared to only using persulfate. During 5 min to 10 min, the isolated Fe on the Fe_78_Si_9_B_13_ ribbon is gradually activated to ferrous by UV-Vis light following with the dominant radical production of ∙OH (2.7 V) for dye degradation (Fig. 7). Subsequently, the SO_4_∙^−^ (2.5–3.1 V) activation begins and becomes the principal reactive species for dye molecule composition. As confirmed by the UV-Vis spectra characterization in Fig. 6d, the dye molecules are finally degraded to non-toxic substances.

In summary, this is the first report of using an amorphous Fe_78_Si_9_B_13_ alloy for activating persulfate to rapidly produce the sulfate radicals under UV-Vis light. In this work, the generated reactive species (∙OH and SO_4_∙^−^) achieve an ultra-fast MB dye degradation within 20 min. The amorphous Fe_78_Si_9_B_13_ alloy shows a superior stability and reusability during the MB dye degradation. Such high stable and reusable catalyst is expected to open a new gate for various potential catalytic processes in hydrocarbon conversion and environmental science.

## Methods

### Materials

The amorphous alloy ribbons were fabricated using melt-spinning methodology under argon protection. Starting from the elemental pieces having the purity higher than 99.9 wt%, master alloys with the nominal compositions of Fe_78_Si_9_B_13_ (in atomic percentage) were prepared by arc melting under a Ti-gettered argon atmosphere. The metallic glassy ribbons of the alloys were prepared in an argon atmosphere by induction melting the master alloy ingots in a quartz crucible and ejecting it onto a single-roller using a melt spinner. The surface speed of the copper roller used was about 30 m/s. The as-quenched ribbons were approximately 5 mm wide and 30–40 μm thickness^45^,^61^,^62^. Sodium persulfate (Na_2_S_2_O_8_) was supplied by BDH Chemicals Ltd Poole (England). The MB dye used throughout this experiment was purchased from Xilong Chemical Co., Ltd (China). The dye aqueous solutions were diluted by Milli-Q water of 18.2 MΩ · cm. A 0.5 M sodium nitrite (NaNO_2_) solution was employed as the quenching agent to prevent further dye degradation. Nitric acid (2% w/w HNO_3_) was used for dissolution and dilution of the treated dye solution for further iron concentration tests. High purity tertiary butanol (C_4_H_10_O) and ethanol (C_2_H_6_O) were used without further purification.

### Characterization and Catalytic Degradation

The structural features of the Fe-based amorphous ribbons at different use times were examined by X-ray diffraction (XRD) in a PANalytical Empyrean diffractometer with monochromated Co-Kα radiation. A Perkin Elmer Lambda 35 UV-Vis spectrometer (Shelton, CT, USA) was employed for recording the UV-Vis diffuse reflectance spectra (UV-DRS) and BaSO_4_ was used as the reference. Scanning electron microscope (SEM), with energy-dispersive X-ray spectroscopy (EDS), on a JEOL 6000 instrument (Japan) was used to characterize the ribbons before and after dye degradation. From the SEM images, the proportion of the formed SiO_2_ layer at various used times was calculated with the software ImageJ. The conductivity of the treated samples was monitored with an Oakton PC 2700 Benchtop Meter (USA). The amorphous Fe_78_Si_9_B_13_ alloy activation of SO_4_∙^−^ from sodium persulfate (Na_2_S_2_O_8_) was observed by the catalytic degradation of methylene blue (MB) dye in a 200 ml beaker containing 100 ml dye solution (20 ppm). The target dye solution was carried out with a Vortex-Genie 2 mixer under the irradiation of a 300 W UV-Visible light (Perfectlight Scientific Pty Ltd, Beijing, China) with an irradiation intensity of 7.7, 11.1, or 14.8 μW/cm^2^. Catalyst dosage of 0.15, 0.5 or 2.0 g/L respectively was added to the dye solution followed by the addition of sodium persulfate (Na_2_S_2_O_8_) (0.5, 1.0, 2.0 or 5.0 mM, respectively) to commence the experiment. Dye aqueous samples were sampled at time intervals of 1, 2, 5, 10, 15 and 20 min followed by further filtration by a 0.45 μm Pall Corporation (New York, USA) filter. The filtered samples were tested in turn with a UV-Vis spectrometer (Shelton, CT, USA) and an Oakton PC 2700 Benchtop Meter for monitoring the dye degradation and conductivities, respectively. Dye degradation efficiency and the corresponding kinetic rates (*k*) were calculated according to Equation 5 and Equation 6, respectively.


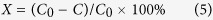


where *C*_*0*_ and *C* are the initial concentration and the concentration at time *t* of MB dye


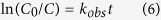


where *k*_*obs*_ is the kinetic rate constant; *C*_*0*_ is the initial concentration of dye; *C* is the dye concentration at time *t*.

The sample solutions were diluted 10 times with 2% w/w nitric acid (HNO_3_) and then filtered by the 0.45 μm filter before the ICP-OES test (Optima 8300 ICP-OES Spectrometer, PerkinElmer). The initial pH of MB dye aqueous solution was reduced from 5.14 to 3.39 after addition of sodium persulfate (Na_2_S_2_O_8_); no further pH adjustment was undertaken. Milli-Q water was first used for washing the used Fe_78_Si_9_B_13_ ribbons in an ultrasonic cleaner for 90 seconds, then the surface was further cleaned by alcohol, after which the ribbons were preserved in the alcohol solution for further characterization.

## Additional Information

**How to cite this article**: Jia, Z. *et al*. Ultra-sustainable Fe_78_Si_9_B_13_ metallic glass as a catalyst for activation of persulfate on methylene blue degradation under UV-Vis light. *Sci. Rep.* **6**, 38520; doi: 10.1038/srep38520 (2016).

**Publisher's note:** Springer Nature remains neutral with regard to jurisdictional claims in published maps and institutional affiliations.

## Supplementary Material

Supporting Information

## Figures and Tables

**Table 1:** Reaction rates (*k*) under various conditions (if not mentioned, the reaction conditions are irradiation intensity: 7.7 μW/cm^2^, dye concentration: 20 ppm, Fe_78_Si_9_B_13_ dosage: 0.5 g/L, persulfate concentration: 1.0 mM).

Parameters		Reaction rates, *k* (min^−1^)	R^2^
persulfate concentration (mM)	0.0	0.002	0.999
	0.5	0.260	0.988
	1.0	0.626	0.995
	2.0	0.640	0.972
	5.0	0.658	0.988
Catalyst dosage (g/L)	0.00	0.092	0.983
	0.15	0.303	0.998
	0.50	0.640	0.972
	2.00	0.736	0.998
Irradiation intensity (μW/cm^2^)	0	0.228	0.992
	7.7	0.640	0.972
	11.1	0.659	0.991
	14.8	0.673	0.999
