# Supplementary material for: Ultra-sustainable Fe78Si9B13 metallic glass as a catalyst for activation of persulfate on methylene blue degradation under UV-Vis light
Source: Sci Rep. 2016 Dec 6;6:38520. doi: 10.1038/srep38520 (PMC5138629; doi:10.1038/srep38520)
Supplement: Supporting Information [file srep38520-s1.pdf]

Supporting Information

Ultra-sustainable $Fe_{78}Si_9B_{13}$ metallic glass as a catalyst for activation of persulfate on methylene blue degradation under UV-Vis light

Zhe Jia[1], Xiaoguang Duan[2], Wenchang Zhang[3], Weimin Wang[4], Hongqi Sun[1], Shaobin Wang[2] and Lai-Chang Zhang [1,*]

[1]School of Engineering, Edith Cowan University, 270 Joondalup Drive, Joondalup, Perth, WA 6027, Australia
[2]Department of Chemical Engineering, Curtin University, GPO Box U1987, Perth, WA 6845, Australia
[3]Environmental Protection Administration of Ji'an City, Ji'an, Jiangxi Province, 343000, China
[4]School of Materials Science and Engineering, Shandong University, Jinan, Shandong 250061, China

[*]Corresponding author: *L. C. Zhang*. Tel: +618 **6304 2322; F**ax: +618 **6304 5811; Email addresses:** l.zhang@ecu.edu.au; lczhangimr@gmail.com

[#] Authors who contributed equally to this study.

[*]Corresponding author. Tel: +618 **6304 2322; f**ax: +618 **6304 5811; email addresses:** l.zhang@ecu.edu.au; lczhangimr@gmail.com

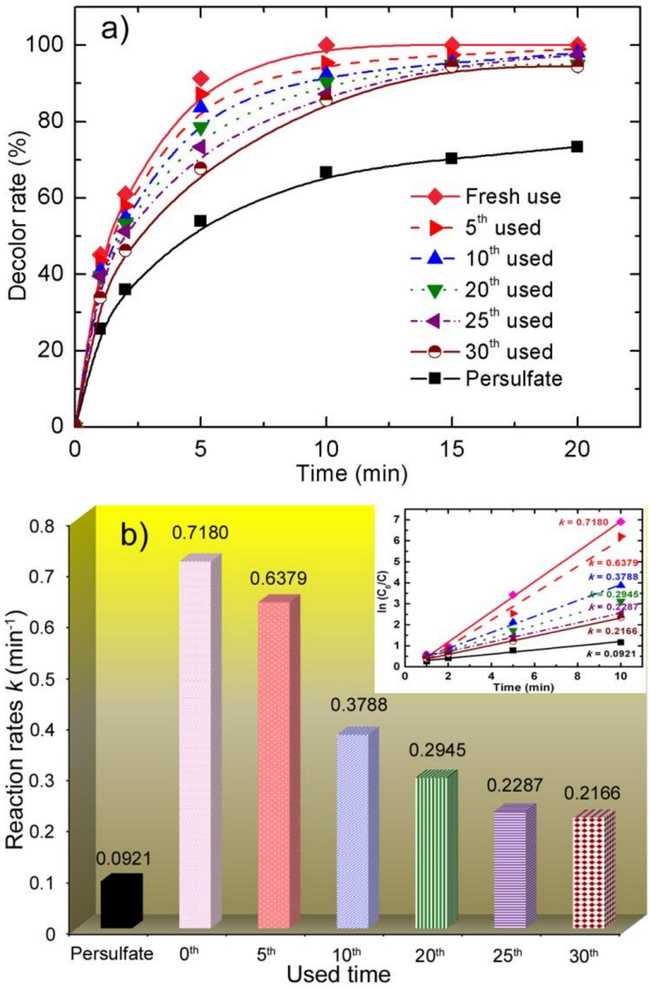

Figure S1. (a) MB dye decolor rates with continuous removal processes by $Fe_{78}Si_9B_{13}$ ribbons, (b) changes of reaction rates k for various used $Fe_{78}Si_9B_{13}$ ribbons, with an inset showing the first-order fit of MB dye degradation.

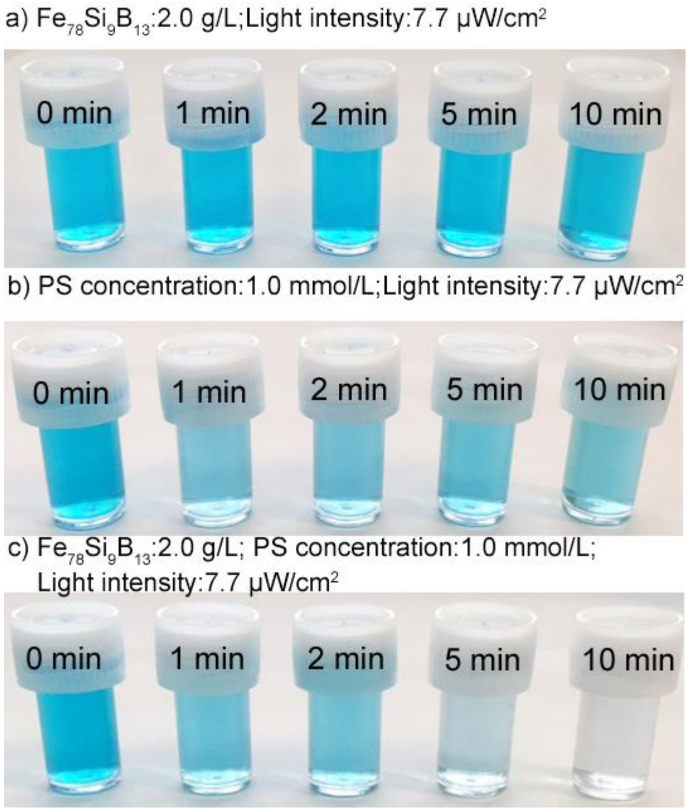

Figure S2. Visible color fading of MB dye concentration of 10 ppm under various conditions.
